# Transylvania Medical Journal

**Published:** 1852-10

**Authors:** 


### TRANSYLVANIA MEDICAL JOURNAL.

THIS journal has been enlarged and improved in its appearance, and is now under the management of Dr. L. J. Frazee, formerly of Maysville, Ky. Dr. Frazee is known to the profession by a small volume which he published on his return from Europe, a few years ago, entitled "The Medical Student in Paris." He is a pleasant writer, a gentleman of attainments, ambitious of eminence in his profession, and, we have no doubt, will edit the Transylvania Journal well. He has our best wishes, and we can venture to promise him that if his new field of labor should not prove profitable in a pecuniary way, it will give him fine scope for intellectual exercise.

